# The return of nature? Negotiating the ‘renaturation’ of the Isar as an envirotechnical landscape

**DOI:** 10.1177/03063127231217577

**Published:** 2023-12-19

**Authors:** Daniel Aditya Tjhin

**Affiliations:** Uppsala University, Uppsala, Sweden

**Keywords:** envirotechnical analysis, sociotechnical imaginaries, renaturation, Isar, environmental history

## Abstract

How can we trace differing normative values, and especially in alternative imaginaries of environmentally sustainable futures? To address this issue, this article extends the sociotechnical imaginaries framework by providing conceptual tools to understand the underlying rationale of alternative environmental imaginaries—through an envirotechnical analysis. I analyse an urban river restoration project called the Isar-Plan in Munich, Germany, where the notion of ‘renaturation’ was at the centre a controversy over designs for the project. By positing the river as an envirotechnical landscape, the normative dimensions of nature, science and technology within environmental transformations can be constructively integrated within co-productionist analyses in science and technology studies. The article shows how existing societal values are shaped by prior systems and regimes, constructing local imaginaries of desirable environmental futures. Envirotechnical analyses also increase our ability to identify differing normative values, and could thus be further applied in cases where the normative assumptions behind opaque notions otherwise would be left underexplored.

In the context of research on global environmental change, Beck et al. (2021) suggest that the framework of sociotechnical imaginaries (STI) may contribute to our understanding sustainability by illuminating collective visions of desirable—or resisted—environmental futures. Empirical examples illustrate how imaginaries of sustainable futures enable or limit the scope and spaces of political action for transformation. Beck et al. (2021) state that the framework of sociotechnical imaginaries looks at environmental transformation ‘not merely as changes in ways of using nature (e.g. agricultural, industrial, exploitative), but appreciating that associated notions of progress … tend to map onto the world only in highly imperfect ways’ (p. 145). They suggest that a major future challenge in addressing global environmental problems is to better understand how ‘alternative visions and transformative practices resonate with existing societal values, political structures and technical infrastructures in which they are embedded’ (Beck et al., 2021, p. 149). Explicating these embedded values, structures, and infrastructures is important for understanding environmentally transformative visions and practices. Because of their empirical focus on environmental transformations, Beck et al. consider this an importantly unmet challenge, despite a number of insightful studies that pay attention to the stabilization of alternative visions of the future (e.g. Felt, 2015; Kim, 2015; Levidow & Raman, 2020).

The interconnectedness between environmental transformations, sociotechnical systems and governance requires a multidimensional engagement. Such attention to additional dimensions can be found outside the framework of STIs, for example, in scholarship with interest in environmental history (Jørgensen et al., 2013). Of specific use is Pritchard’s (2012) study of the mutual constitution of science and technology, environmental management, and power in 19th-and 20th-century France. Her envirotechnical analysis illuminates how environmental knowledge and governance are integral to the constitution of power—or perhaps a constitution of a powerful ‘imaginary’, using the STI vocabulary. The relation between environmental knowledge, governance and power, translated into transformative practices and effective policies, provides a more in-depth understanding of the transformed environments of the former French colonies (Pritchard, 2012).

Pritchard (2013) calls for a dialogue between environmental history and science and technology studies (STS). Rowland and Passoth (2015) echo this call by suggesting for more engagement with envirotechnical analysis in STS inquiries into environmental politics. The cross-disciplinary conversation that they call for is also the point of departure for this study. In her book *Confluence*, Pritchard (2011) conceptualizes and mobilizes envirotechnical analysis to explicate how technological development and environmental management reciprocally constitute political power and nation-building, studying nature and technology both discursively and materially. Envirotechnical analysis strengthens the STI framework, especially through its attention to the dynamic relationships between nature, technology, and society, in their material manifestations over time (Pritchard, 2011).

To explore how envirotechnical analyses engage with visions and practices of environmental transformations, this study turns to an urban river restoration project in Munich, and uses the Isar-Plan as an illustrative example. Primary sources are municipal draft resolutions from 1984 and from 2005, along with newspaper articles and governmental pamphlets during the restoration period from 2000 to 2011. Secondary sources are primarily from the German anthology series on the Isar compiled by Ralf Sartori (2010a, 2011b) who had documented interviews with and writings from the actors involved with the Isar-Plan. Additionally, the works of Julia Düchs (2014) and Frédéric Rossano (2016) serve as secondary sources along with their own interpretations of the Isar-Plan. Most sources are in German, translated here by the author.

The Isar is an alpine river and the fourth largest river in Bavaria, flowing through major and minor urban centres, most notable Munich. Munich’s relationship with the Isar has been important for developments in the city since the 12^th^ century, and even though its relationship has changed over the centuries the Isar is still important to Munich for its ecological and recreational values. The Isar-Plan sought to redesign the river under the banner of ‘*Renaturierung*’, or renaturation. Eventually the notion of renaturation would become increasingly problematic, as it was unclear what the Isar renaturation would entail. It was implicitly understood to be coupled to restoration efforts that would recreate a ‘natural’ Isar, seemingly free from human machinations, yet still within existing technological constraints. The restoration project was contested by residents of Munich, because of their ideas of a ‘renatured’ environment—eventually affecting the development of certain sociotechnical systems in the sought-after environmental transformations.

I present a narrative of the Isar-Plan from its conception to completions, with particular focus on how Isar’s renaturation was conceived, destabilized, and eventually re-stabilized after the project’s completion. Here I highlight how Isar’s renaturation was negotiated between different actors, and how these negotiations were materially constrained by existing infrastructures. Finally, I conclude by reflecting on this study’s main contributions and limitations, and how the envirotechnical approach provides a more multidimensional engagement in analysing environmental transformations.

## STI for alternative environmental transformations

There has been no shortage of analyses of sociotechnical imaginaries ever since the framework’s formulation (see also Jasanoff & Kim, 2009, 2015); the abundance sparked an accidental special issue of this journal on STIs (Sismondo, 2020). Not only is the framework frequently applied, but it is also used to develop new concepts such as future essentialism (Schiølin, 2020) or crisis imaginaries (Kalmbach et al., 2020). The framework is popular outside STS as well, for example in energy research (e.g. Hess & Sovacool, 2020). The richness of studies of STIs clarifies how malleable the framework has become—seamlessly applied within various disciplines, assumedly without losing its analytical value. It is of little wonder then, why the concept of imaginaries has grown in popularity: conceived as a holistic framework, yet permissive enough to be mobilized in relation to all sorts of phenomena.

Studies of STIs have put a lot of emphasis on alternative visions, usually contrasted with global or top-down imaginaries. Bringing together a set of empirical case studies, Mager and Katzenbach (2021) assert that STIs are often multiple and contested, rather than monolithic and linear. Andersson and Westholm (2019) reveal how conflicting interests—between biodiversity and industrial productivity in their case—are managed as a matter of finding compromises between competing future images and values. Levidow and Raman (2020) study how the ecomodernist policy of UK’s low-carbon waste-energy futures combined dominant and alternative imaginaries, where the policy gained authority and acceptance while softening tensions between future visions. Kim (2015), in his analysis of South Korea’s engagement with issues to do with science and technology, claims that Korean NGOs and lay initiatives have failed to gain success due to the lack of concrete alternative imaginaries. The lack of alternative imaginaries in South Africa is another example, where Cecil Rhodes was able to enact his imperialist vision; this transformed the environment of South Africa—both literally and politically—without regard to previous and existing local conditions (Storey, 2015). In contrast, Felt (2015) has argued that alternative imaginaries of technology managed to stabilize in Austria, creating an enduring sociotechnical imaginary, due to a gradual, long-term process of national identity formation.

The studies listed above show that there is no dearth of research within the STI framework that might be of importance to Beck et al.’s (2021) envisioned challenge in understanding alternative imaginaries of sustainable futures. What is more peculiar in their case is the focus on environmental transformations, and indeed most of the studies mentioned above do not explicitly deal with transformative practices in relation to the environment. Thus, despite the various insights, there is potentially still a lot of merit in complementing STI analyses of environmental futures with insights from environmental history. Here, I propose that engaging with envirotechnical analysis can provide detailed understandings on how environmental transformations are enacted materially and discursively. The envirotechnical approach (Pritchard, 2011) provides conceptual bases for accounting for existing societal values, political structures and (enviro-)technical infrastructures—with explanatory power for understanding why such values, structures and infrastructures exists within a particular society.

## STS and the envirotechnical approach

From the framework’s theoretical development (Pritchard, 2011) to the more general call for a cross-disciplinary conversation (Pritchard, 2013), envirotechnical analysis is not at all foreign to STS. For Rowland and Passoth (2015), the aim of envirotechnical approach is to add ‘nature’ as a concept to be studied by STS, alongside science and technology. As such, envirotechnical analysis is of value not only to those engaged in studying environmental politics, but also to those studying infrastructure and the state—Rowland and Passoth (2015) see Pritchard’s (2011) work as an extension to Jasanoff’s (2004) idiom of co-production. Despite the potentials and compatibilities, there has been little explicit engagement of the framework by STS scholars. As questions on environmental transformations and politics seem to become ever more pressing (Beck et al., 2021; Fisher et al., 2022), it is timely for STS scholars to engage further with envirotechnical analysis.

Envirotechnical analysis works through three interconnected levels—descriptive, historical and conceptual (Pritchard, 2011). The first level describes how social groups interact with their environment over time. The second level explores the historically dynamic relationship between technology and environmental management, with attention to political identities and state building. In this relationship, ideas about nature and technology become intertwined with national or local identity, immanent within technological systems and natural management. Lastly, in order to conceptualize nature, technology and their relations ‘within’ and ‘as’ history, Pritchard (2011, p. 3) develops two concepts: envirotechnical systems and envirotechnical regimes. The conceptual tools put emphases on the historical actors and analysts’ categories of nature and technology, as well as the ecological dimension of supposedly ‘technological’ artefacts.

Taking a constructivist stance, ideas of nature and technology are not self-evident and are contingent. Rather than positing nature and technology as a dichotomous pair, envirotechnical analysis suggests that nature and technology are both material and discursive, and that their constructions need to be unpacked simultaneously. Rejecting easy, straightforward definitions, envirotechnical analysis is sensitive to the underlying assumptions in invocations of nature and technology as concepts that inform decisions and coordinate actions.

Envirotechnical systems are historically and culturally specific configurations that are at the same time ‘ecological’ and ‘technological’ (Pritchard, 2011, p. 19). These systems are composed not only of material artefacts, but also of practices, people, institutions and ecologies. Thus, they encompass not only ‘nature’ and ‘technology’ but also their social, cultural and political dimensions. ‘Systems’ are in the plural due to the diverse forms the systems might take historically, and because several may coexist or compete. This plurality points to how environments and technologies continually reshape either individual parts of envirotechnical system or the whole.

Whereas the concept of envirotechnical systems is mobilized for descriptive analyses of existing systems, the concept of envirotechnical regimes is the complementary prescriptive formulation (Pritchard, 2011). It is ‘the institutions, people, ideologies, technologies and landscapes that together define, justify, build and maintain a particular envirotechnical system as normative’ (Pritchard, 2011, p. 23). The metaphor of ‘regime’ brings attention to the politics of envirotechnical systems and highlight the underlying power relations behind the development, implementation and use of systems. Taken together, the concepts of systems and regimes have potential to ‘bring all the vital insights of social, cultural, and political studies of technology to envirotechnical analysis while incorporating nature into their inquiries in a reflective, critical way’ (Pritchard, 2011, p. 24).

In addition to these conceptual ambitions, recent publications using the envirotechnical approach have further illuminated how such analysis might look like. Mittlefehldt (2018) unpacks how renewable energy technologies reinforce and entrench historical inequalities within issues of environmental justice. Ruuskanen (2018) shows how different framing of peatland have led to different sorts of knowledge production and policy decisions. Pritchard (2017) argues against the problematic framing of problems and knowledge in NASA’s *City Lights*, which may reproduce old conservation rhetoric. In narrating the envirotechnical history of Aktau’s nuclear landscape, Guth (2022) takes inspiration from STI to illustrate how Kazakhstan’s visions of atomic-powered future are marred by prior interventions. Lastly, MacFarlane (2020) argues that the Niagara Falls is a case of what is called a ‘disguised design’, where engineers blends steel and concrete with water, rock, weeds and ice to preserve a scenic nature.

## The Isar, an envirotechnical landscape

Munich and the Isar have shared their history since at least the 12th century. Munich, originally a simple Benedictine monastery, only rose to prominence as a trade node due to the existence of a toll bridge (Winiwarter et al., 2016). This toll bridge was a result of a political intrigue of the then Duke of Bavaria—Duke Henry the Lion—who had burned a neighbouring toll bridge owned by Bishop Otto of Freising. This small glimpse into Munich’s early history already exemplifies how the Isar was an envirotechnical landscape from the very beginning, where the importance of both the city and the river was forged by a system (the toll bridge) and regime (the duke’s political interest). As Winiwarter et al. (2016) have shown, the Munich-Isar relationship would only increase in importance, culminating in a city traversed with water canals with a total length of about 72 kilometres. The canals, sourced from the Isar, were multi-functional: they were at the same time a freshwater source, a transport system and a sewage system (Winiwarter et al., 2016). It was not until the 19^th^ century that Munich started to gain independence from the Isar as a freshwater source. A combination of epidemics, alternative sewage and drinking systems, and increased municipal autonomy resulted in an increasingly canalled river for urban expansion.

As Munich was stripped of its status as a ‘fortress city’, it started to incorporate the east side of the Isar as part of its urban expansion through the development of canals (München, 1986). Work was done to straighten the Isar and the adjoining Isar area was envisioned as a new building area (Winiwarter et al., 2016). A risk of this method of urban expansion was flooding—which would become a constant concern for Munich in the 19^th^ century, because the Isar would flood once every three years (Winiwarter et al., 2016). The result of centuries of efforts to control floods to accommodate rapid urban expansion and eventually industrialization caused a complete change of the Isar’s course and the loss of the original river landscape (München, 1986). However, the drives for urban expansion and industrialization were not the only reasons for the canalling of the Isar. In the late 19^th^ century, Munich followed the engineer Johann Tulla’s principle on which geometry and functionality were the sought-after qualities of a beautiful river (Rossano, 2016).

The rapid urban expansion and industrialization consequently increased the need for energy generation (Renner, 2011). Hydropower was considered suitable to fulfil this need, especially after the first world war (Landry, 2015; Renner, 2011). Four hydroelectric power plants were built in Munich along the river between 1899 and 1924 (München, 1986), and parts of the upper Isar were dammed to accommodate for the Walchensee hydroelectric power station (Landry, 2015). By the second half of the 20^th^ century, there were more than 30 hydroelectric power stations along the entire Isar stream (Renner, 2011). As the power plants needed a certain amount of water to effectively generate power and they had legal rights to divert the Isar’s streams (München, 1986), they significantly affected the water level of the river (Renner, 2011). Despite the environmental degradations resulting from these systems, the unconditional belief in progress allowed only little resistance to the implementations of the power plants (Renner, 2011). The power plants and all the practices undertaken to facilitate their operations were then material manifestations of this very specific ideology of progress, further legitimizing the authorities and interests of the regimes.

## The idea of a beautiful nature

While the urban environment in Munich underwent sweeping changes, rapid industrialization in Germany incited a response in which nature became one of art’s grand topics (Hölzl, 2006). This phenomenon also coincided with another: tourism (Hölzl, 2006). Poets, painters, and other artists created the impression of a sublime and romantic nature untouched by human machinations, with the Alps standing as a prime example of wild and untamed nature (Hölzl, 2006). Meanwhile, tourism rapidly transformed the Bavarian Alps into Munich’s front yard for weekend tourists; by 1900 the Alpine nature had its appearance transformed with massive hotels, souvenir shops, railways, log cabins and cable railways (Hölzl, 2006).

From the romantic period to the turn of the twentieth century, the invention of a sublime and romantic nature became ever more present in broader circles of society and became a major aspect of the cultural formation of society (Hölzl, 2006). The constructed ‘nature’ can be exemplified by ‘natural’ mountain ridges immaculately manicured with hiking paths (Hölzl, 2006). Thus, the idea of nature distinct from human and technology is flawed from its conception: Nature as a no man’s land did not imprint its cultural significance without human exploitation. However, many works of art performed visions of pristine nature by taking technoscientific elements out of the picture—often literally. The romanticists’ ideals of untouched nature prefigured the actions of early Bavarian environmental organizations (Hölzl, 2006).

As a particularly aesthetic notion of nature took hold, it became a foundation of various nature protection organizations in Bavaria that would eventually be involved as part of the Isar’s envirotechnical regimes (Hölzl, 2006). Often, by mobilizing public opinions and formal petitions, they resisted projects that transgressed the limits of ‘natural’ areas. In the context of Munich’s fervour for urban expansion and industrialization, the arguments of Bavarian environmental organizations had to balance utopian ideals of nature with engineering expertise and needs. Although the organizations had some success in putting environmentalism within the strict agenda of modernization, they also were the target of criticisms from the media and parliaments (Hölzl, 2006). Additionally, their attempts to suggest a more careful path of modernization often ended in failure, because their efforts depended entirely on ‘the goodwill of the administration’—political decisions were negotiated in a small, homogenous and elitist circle (Hölzl, 2006, p. 37).

## The narrative of ‘renaturation’ in the Isar-Plan

The precursor to the Isar-Plan was a 1984 motion of Munich’s city council titled ‘Nature in the City’ (München, 1986). The motion called for care and renaturation of wetlands, the Isar amongst them. It pointed out that the Isar in Munich’s urban area had lost its wild Alpine character due to canalling, energy production, and bank fortification, and that it would be appropriate to bring out the natural character of the Isar as much as possible. In 1985, the mayor, along with the planning department, the environmental protection department, the building department, and independent landscape architects, were commissioned to develop a concept for protection, restoration, conservation and development of the Isar floodplains—which would eventually be called the ‘Isar-Plan’ (München, 1986). The city council considered the Isar restoration project to be crucial as its floodplains were considered the most important landscape of Munich not only in terms of ecological and climate factors, but also because of its quality as a recreation area.

Even though discussions continued until 1986, a resolution from the committee for urban planning and building regulations showed that restoring the Isar was no small feat, mainly due to existing envirotechnical systems in place, such as hydroelectric power plants (München, 1986). The diversion of the Isar’s streams to the power plants frequently dried the urban Isar for months (Düchs, 2010) and as the power plants had legal rights to divert the Isar’s streams, there was opposition to plans to raise the water level. The power plant operators only agreed to the plans once thorough feasibility studies were done (Sartori, 2011c). Walter Binder, head of the department of water development in the Bavarian state office, remarked that the power plant operators acted as if they owned the Isar streams, even though it was clearly a common good (Sartori, 2010b). Despite the lively discussions until 1989 on the Isar’s restoration, plans to renovate the Isar floodplains were ultimately shelved because the stakeholders thought that ‘the time was not yet ripe’ (Renner, 2011, p. 92).

In 1995, the Isar-Plan working group was finally created. The working group consisted of the Bavarian state, represented by the *Wasserwirtschafstamt* (Water Management Office, from here on WWA), and the city of Munich, represented by the building, planning, health and environment departments—under the leadership of the department for urban planning and building regulations (Düchs, 2010). The two actors within the working group had different agendas and roles, as Düchs (2010) described: The Bavarian state financed 55% of the project and was mostly concerned with Isar’s function as a wild Alpine stream for ecological and nature protection purposes. The city of Munich, meanwhile, financed 45% of the project and was responsible for the construction of the project and the accommodation of suggestions from the citizens. Their main concerns were flood protection and technical feasibility of the project, where aesthetic improvements were considered as welcome side effect. Together, they set up three objectives for the Isar-Plan: improvement of flood protection, ecological quality, and recreational possibilities (Sartori, 2010b).

In addition to the working group, the activist group Isar-Allianz and the local district committees of the project area were also involved in the Isar-Plan (Düchs, 2010). Isar-Allianz brought heterogeneous expertise and experience from a previous renaturation project. It considered itself the ‘ecological conscience’ of the Isar-Plan (Düchs, 2010, p. 74), and its aim was to incorporate more nature into the design of the river. District committees represented the voices of local residents who lived close to the project area, who needed assurances that the renaturation would improve recreation instead of commercial opportunities (Düchs, 2010). Sartori (2011a, p. 73), however, remarked that the activist group and the district committees were ‘not directly represented in the working group, not authorized to make decisions, not informed and not listened to’ as they only had technical meetings with the working group once or twice a year. Nikolaus Döring, one of the founders of the Isar-Allianz, stated that the working group often claimed as its own improved initiatives from public engagement, thus acting as if it did not want to encourage further public participation or questions regarding its expertise (Döring, 2010).

One result of such public engagement was the eventual prominence of renaturation within the project itself. The term ‘renaturation’ was only used once in the original formulations of the motion (München, 1986)—which was unsurprising, as the notion of ‘renaturation’ was relatively new in Germany and was only starting to become more commonly used in the late 1980s (Düchs, 2010). Moreover, it was used to describe very different projects, usually ones where ruins would be replaced with a ‘*naturnah’* (‘close-to-nature’) condition (Düchs, 2010). Hence, the original plan from the working group to restore the Isar was simply to raise the levee and build more walls (Döring, 2011). The Isar-Allianz fought vehemently against the proposal. It had to prove, through models and presentations, that renaturation of the Isar floodplains was not simply an utopian vision (Döring, 2010, 2011). In the end, it successfully coupled flood protection with the notion of renaturation, after three years of constant negotiations (Döring, 2011). Still, Döring (2011) wrote that the Isar-Allianz would have failed if not for the mayor’s personal support during the working group meetings—reminiscent of early Bavarian conservationists efforts (Hölzl, 2006).

After a period of inspection and negotiations, the project finally started in 2000, encompassing an eight kilometre stretch from the southern city border to the inner city (Arzet & Joven, 2008). Düchs (2010) noted how some of the early renaturation measures had remarkable descriptions that evoked an image of liberation and vitality—as if the Isar was a ‘patient’ to be healed and be given a new lease of life so it could flow wildly once more (p. 71). Some local residents were not so keen about renaturation, however, fearing a gentrification of the living area (Düchs, 2010). The working group managed to assuage such concerns through public talks, flyers and guided tours (Düchs, 2010). In addition, the image of liberation and vitality was echoed by local media, saying that the renatured Isar was ‘wilder, faster, stronger’ (Wolff, 2001), in effect a ‘wild river’ (Schmidt, 2002). In the first years of the Isar-Plan, the media kept reinforcing the apparent success of the renaturation through three images: ‘the river’s corpse has recovered’, it was ‘freed from corsets’ and ‘it rages and lives’ (Düchs, 2010, p. 71).

## The urban Isar and its complexities

The Isar flows ever northward, to the middle of Munich’s dense urban area. The renaturation of the urban Isar was considered to be the most technically challenging part of the project (Sartori, 2011a). Klaus Arzet, head of WWA, stressed the importance of differentiating the urban and non-urban parts (Sartori, 2011c). For Arzet, the southern part of Isar could become as close-to-nature as possible, but renaturation of the urban Isar had a lot more constraints (Sartori, 2011c). The constraints were not only functional ones, such as energy generation or recreation facilities, but also the multitude of quay walls, buildings, and bridges that were culturally significant monuments under protection (München, 2005). Hence, a renatured urban Isar had to be visually pleasing, functionally sound and accessible for recreation under these limitations (München, 2005).

Due to these challenges, the WWA launched an interdisciplinary landscape design competition in November 2002. The competition blurb stated that the design had to improve the ecological quality of the riverbed, diversify stream structure and restore sediment transport, while also facilitating recreational uses in an aesthetically pleasing nature-like landscape, and enhancing the cultural value of the Isar landscape (Rossano, 2016). Important to note is that the WWA did not mean that the design had to be ‘naturalistic’; Arzet, for example, wanted more variety in the designs and highlighted the appeal of the river’s urban character (Sartori, 2011c). The competition participants were anonymized (Sartori, 2011c), with state and non-state actors of diverse expertise as jurors (München, 2005).

**Figure 1.**
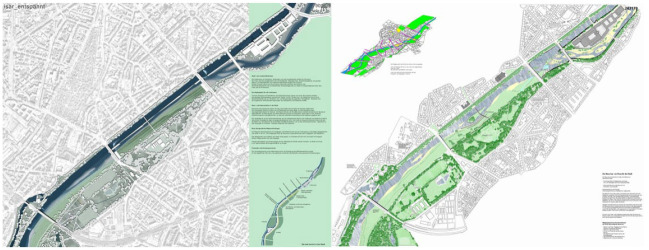
Left: Irene Burkhardt’s design and right: Winfrid Jerney’s design (München, 2005).

The results of the competition were announced to the public in April 2003. Of particular interest were the first and second place designs, by Irene Burkhardt and Winfrid Jerney respectively (Figure 1). Burkhardt proposed widening the riverbed and dividing the river course with the addition of a side-channel parallel to the main course—separated by sloping and partially submerged concrete slabs (München, 2005). The side-channel would establish ecological continuity from and to the main course. The juries were impressed with the design quality to ensure flood protection while meeting hydrological requirements—additionally, a recreational water landscape possibility was convincingly met (Düchs, 2014). Despite the still canalled look of the river and the reduction of flood meadows, the jury argued that it was not a disadvantage because usable water areas were offered through the side-channel. The preservation of the quay walls was also deemed unproblematic, and the jury stressed the advantages of the design’s recreational values. Economically and technically, the implementation of the design was deemed uncomplicated, adding to its hydraulic and recreational achievements. The virtue of Burkhardt’s design was its ability to improve flood protection and ecological qualities without the need of radically changing its existing form.

The second-place design by Jerney also proposed a generous widening of the riverbed, but with new islands in its middle to create new side-channels. Existing weirs would be demolished and replaced with an integrated fish pass, providing a biological consistency between Isar and its side-channels (München, 2005). Jerney’s design gave an impression of wilderness free of technical constraints, due to hidden quay walls. The jury praised the improvement of Isar’s hydrological features and aesthetics, and the plan was deemed economical and easy to implement (Düchs, 2014). They spent a lot more focus on the design’s aesthetic values: how the river became more perceivable with the design, and how free view of the water was restored. Little to nothing was said about the design’s effects on flood protection, nor on recreational values outside of aesthetic improvements—in contrast with the commentaries Burkhardt received.

## A controversial decision

The publication of the competition’s results marked the start of the Isar-Plan’s controversy, centred around the notion of ‘renaturation’. Both state and non-state actors voiced their dissatisfactions with the published results. Part of the city council and district committees saw no improvements in Burkhardt’s design (Schleicher, 2003), and the citizens could not agree with the decision from the jury. The public described the first design as ‘banal’ and a ‘concrete monster’, rejecting it with a slogan of ‘*Renaturieren statt Betonieren’* (renaturating instead of concreting) (Schleicher, 2003). Many considered Burkhardt’s design to run counter to the actual intention of renaturation (Sartori, 2011c). Arzet described the whole situation as ‘unfortunate for everyone involved’ especially as it could have jeopardized the whole project (Sartori, 2011c, p. 76). The situation was complicated by the legal and monetary rights given to Burkhardt, as the first-place winner, to implement the design. If the state were to give the planning contract to someone else, it would have to compensate Burkhardt and run the risk of being sued for damages (München, 2005). The state decided to carry out a mediation process with a roundtable discussion (Arzet, 2004; München, 2005; Sartori, 2011c)

The roundtable discussion in July 2004 brought together interest groups (representing, e.g. athletes, fishermen, recreation, nature protection, and culture), district committees, the WWA, the city of Munich, the two prize winners and one external mediator (München, 2004). Prior to the roundtable, the participants had been given two weeks to assess the designs and give alternative suggestions if necessary. Although the interest groups and the district committees assessed the two designs as equal in terms of flood protection, they were overwhelmingly in favour of Jerney’s design: ‘the outcome of the discussion is clear’ (München, 2004, p. 41). Common remarks in their assessments praised Jerney’s close-to-nature design with the layout of a wild river landscape in a metropolitan city, and criticized Burkhardt’s design as monotone and urbanized (München, 2004). One interest group even remarked that Jerney’s design was the only one that ‘seriously corresponds to the idea of renaturation’ (München, 2004, p. 34). Burkhardt commented that the discussions surrounding the Isar’s renaturation had devolved into arguments about taste instead of technical fact (Näger, 2004).

Although the city council had the final say in the decision, the verdict from the roundtable led to a compromise between the two prize winners. The working group divided the urban Isar intro three parts, where Burkhardt would work on the south-middle part, Jerney the middle part and then they would work together for the northernmost part (Arzet, 2004; Näger, 2005a). A final design was finally agreed upon in March 2005, delaying the project by about two years. However, the compromise did not really end the controversy. Parts of the public felt the final preliminary design did not implement previously agreed-upon suggestions. Döring and Heinz Sedlmeier, both part of Isar-Allianz, voiced their displeasure with the design’s biological consistency and the half-measures taken to renature the Isar (Näger, 2005b). They were also critical of the working group’s lack of continued public engagement, as the administration ‘once again sat down in quiet little room’ to finalize the plans (Näger, 2005b). The critique seemed to be vindicated in December 2005, when the working group once again changed the already agreed plan from March (Eisenack, 2005). The new design looked remarkably straight, which prompted further criticisms from the district committees. Representative of the WWA responded to the criticisms by claiming that the new design was needed for the river’s ecological continuity, as it incorporated lessons learnt from the summer flood of August 2005 (Eisenack, 2005). The representative went on to say, ‘We cannot make the Isar so wild and so close-to-nature any longer. The Isar must be more well-behaved.’

## Ending a controversy: A close-to-nature river

Despite some criticisms, the final plan was ratified in March 2006 by both architects, then construction works resumed and the controversy was considered settled. The renaturation project also began to garner recognition locally and internationally. In 2007, the Isar-Plan won the national river basin management award for its exemplary measures in implementing close-to-nature design in an urban setting (Arzet & Joven, 2008; Düchs, 2010). Internationally, the project was visited by water engineers from Brazil, China, and Los Angeles (Düchs, 2010). It was presented as a ‘successful inner-city river landscape project’, claiming to have successfully combined flood protection and urban recreational values with a close-to-nature design (Arzet & Joven, 2008). Even before its completion, the new Isar was said to flow like a wild and natural river, proof of how successful renaturation projects can become (Dürr, 2008). Arzet and Joven (2008, p. 11) wrote that ‘The vision of the river Isar in the 21^st^ century is not the original pre-alpine river landscape, but rather a river that reflects its alpine origin.’

Despite these local and international recognitions, there was still lingering discontent with the extent of Isar-Plan’s renaturation efforts. Sartori, in his interview with Arzet in 2011, kept inquiring about possible further renaturation efforts (Sartori, 2011c). For Sartori, the urban Isar still had too many signs of concrete, and the river’s course was too straight after the so-called renaturation. In turn, Arzet defended the project, saying that the urban Isar was also reconstructed to be close-to-nature, in a more functional sense rather than aesthetical, and that the river must be given time before any further renaturation efforts. ‘We do not have to do everything ourselves’ he claimed, referring to the river’s capability to develop its own course within certain limits (Sartori, 2011c, p. 84).

In another interview, Burkhardt reflected on the controversy and the unexpected reactions her design received (Sartori, 2011d). She hoped that after eight years people could finally be more objective and open in discussing the controversial decision. The controversy, for Burkhardt, boiled down to the fact that nobody knew what ‘renaturation’ or ‘close-to-nature’ would entail for urban Isar, especially as a natural-looking river would not necessarily mean one of high ecological quality (Sartori, 2011d). Burkhardt stated that her design had successfully highlighted the appeal of urban-nature contrasts, but it was ‘hastily condemned’ by the word ‘concrete’, resulting in negative public reactions (Sartori, 2011d, p. 128). For Burkhardt, the compromise plan was well and truly a compromise (Sartori, 2011d), as some of the renaturation ideas tried too hard to look natural without offering clear benefits (Rossano, 2016; Sartori, 2011d).

Lingering discontents aside, a pamphlet, *Der Isar-Plan*, published by the government in 2011 recounted the story of the project, with a surprising amount of revisionism. The Isar prior to the mid-19^th^ century was retroactively called a ‘wild river’ and its canalling was marked as the start of its ‘cultural landscape’ (München, 2011a). In another pamphlet, *Neues Leben für die Isar* (New life for the Isar), the new Isar was described as a ‘close-to-nature river landscape’ (München, 2011b). Most articles published after the Isar-Plan’s completion promoted its renaturation as a success, glossing over the constant contestation of renaturation during its planning (Düchs, 2014). The Isar-Plan was promoted as a renaturation project with close-to-nature river landscape as its results: intact nature and attractive scenery, and a suitable habitat for indigenous river plants and animals (Düchs, 2014). Public receptions of the new Isar were also generally positive: Most considered it as an improvement of their quality of life and a source of inspiration (Ruhland, 2011). The various publications had a lot of discursive conceit embedded within them, however: The Isar was said to ‘have returned into being a river’ and its future ‘lies at the hand of nature’ (München, 2011b), implying that the former Isar was not exactly a river and that its future would be free from human machinations.

Generally, the final consensus was that the Isar-Plan was a success, able to put earlier doubts and controversy to rest. Its program of renaturation was deemed fulfilled, and the new Isar is a ‘win for everyone’ (München, 2011b). However, should one investigate the new Isar further, it holds more similarities to its prior form than might be expected. In terms of river morphology, the mimicry of a natural stream brought modest effects (Rossano, 2016; Sartori, 2011d). Shore variability was as limited as before, and occasional alluvial materials had to be mechanically redistributed by bulldozers after flooding (Rossano, 2016). Rossano (2016, p. 7) called the new Isar a ‘man-made channel in drag’, due to its urban uses and constraints; it had a form similar to that of a wild alpine river but without its performative characteristics. Despite his critical remarks, Rossano (2016) still considered the Isar-Plan as a spectacular achievement in its ability to accommodate both extreme floods and intense urban uses. He considered the Isar landscape to have transcended romantic formalism as an open space without predefined use or program: It is not a flood zone, a regulated park, nor a nature reserve, but is instead a hybrid space for all these together. In other words, the Isar had become a fitting envirotechnical landscape for existing systems and regimes, a result of a decade-long negotiation in a quest for a natural landscape.

## Negotiating the envirotechnical

The case of the Isar-Plan shows intertwined relations between science, technology, politics, and concepts—both materially and discursively. The negotiations for renaturation in the Isar-Plan were indeed materially constrained by existing envirotechnical systems, yet these constraints were considered in varying framings and degrees (see also Pritchard, 2017; Ruuskanen, 2018). The notion of renaturation inadvertently provoked public engagement because of differing visions of what a renatured Isar would and could be. Prior norms of nature (Düchs, 2010; Hölzl, 2006; Rossano, 2016; Winiwarter et al., 2016) led to alternative visions of environmental transformation that could be politically mobilized. ‘Nature’ as a political, material-discursive category cannot be naturalized (Pritchard, 2011; Rowland & Passoth, 2015).

The dissatisfaction of the Isar-Allianz and the riposte by the WWA about possible renaturation measures was a contest between knowledge claims and power relations from different envirotechnical regimes. The Isar-Plan as an urban river restoration project illustrates how an environmental transformation brought various envirotechnical regimes together to assert their normative values—appealing to science, technology and nature in their negotiations. In these negotiations, the importance of prior systems and regimes came to matter as they held norms of what was (im-)possible. The idea of a wild and beautiful nature complicated the negotiation, and recreating this ‘nature’ became the mission statement for the activist group. This fixation on nature was itself constructed, a reaction to industrialization in the age of ‘classical modernity’ (Hölzl, 2006), and thus was not separate from thinking of the Isar as an envirotechnical landscape.

The case of the Isar-Plan is reminiscent of MacFarlane’s (2020) notion of disguised design, where technologies are meant to be unseen through their mimicry of natural appearances. If the engineers of Niagara Falls used ‘as near as may be’ as a shorthand to mask their uncertainty in hydraulic models (Macfarlane, 2020, p. 113), the ‘close-to-nature’ saying was also used to justify the extent of renaturation by putting ‘nature’ and ‘urban’ on an illusory scale (Düchs, 2014). The Isar-Plan working group believed that it had created an urban river superior to its predecessor, just as did the engineers of Niagara Falls (Macfarlane, 2020). However, if the ambition of United States and Canada was to transform the Niagara Falls into hydroelectric complexes, the issue of the Isar-Plan came almost from the opposite direction. It was not the intent to materially ‘disguise’ technology beneath nature, but to get as close-to-nature as possible—although the project ended with a ‘light-green’ solution (Pritchard, 2011, p. 239). The Isar-Plan working group genuinely believed that it had achieved an appropriate mix of wilderness and civilization with its new Isar, in its quest to ‘return’ an envirotechnical complex into its ‘natural’ state. In the end, the legitimization that the project received through prizes, international recognitions, and local media naturalized the renaturation of the Isar-Plan itself (see also Pritchard, 2011).

The focus on renaturation complements studies that have focused on technological progress and commodification as the driving ideologies in environmental transformations (Guth, 2022; Ruuskanen, 2018). Technological progress and commodification were also the driving ideologies for Isar landscape development (Hölzl, 2006; Renner, 2011; Rossano, 2016; Winiwarter et al., 2016). Hence, even in a restoration project that was guided by renaturation, with an intent to move away from existing systems, notions of progress and commodification still entered into the negotiations (Döring, 2011). Concrete walls were taken as the material manifestations of these old ideologies, hence the strong rejection of its appearance in the supposedly renatured river. The case of the Isar thus further illustrates the difficulties of moving away from prior norms in environmental politics, with risks of further perpetuating them (see also Mittlefehldt, 2018), especially if guided by notions that are open for interpretation (e.g. sustainability). It also shows that political participation is never a given, and has to be fought for even in supposedly democratic societies.

## Conclusion: Alternative visions of environmental futures

This article has addressed the challenge made by Beck et al. (2021), by showing how the envirotechnical approach complements co-productionist analyses of environmental politics in times of global environmental change. The conceptual tools of envirotechnical analysis can guide an analysis that aims to trace and understand existing societal values, political structures and technical infrastructures that are both material and discursive. Environmental transformations entangle the normativities of science, technology, and nature, seen as political categories that are both material and discursive.

Approaching the Isar as an envirotechnical landscape with various systems and regimes offers a multidimensional engagement that shows ‘the connections between visions and values attached to sustainable futures and the politics of knowledge brought to support them’ (Beck et al., 2021, p. 149). Here, one of the more important insights offered by the case of the Isar-Plan is how normativity of ‘nature’ becomes important in times of environmental transformations. Thus, as an extension of the co-production idiom (Rowland & Passoth, 2015), Pritchard’s (2011) suggestion that both technology and nature are both material and discursive conveys deeper analysis of ‘nature’ and ‘technology’ as categories. In contrast to STI-inspired analyses that emphasize alternative imaginaries (Andersson & Westholm, 2019; Felt, 2015; Kim, 2015; Levidow & Raman, 2020; Mager & Katzenbach, 2021; Storey, 2015), ‘nature’ as a political category gains a lot of importance in governing environmental transformations. Envirotechnical analysis thus provides conceptual tools for STI and co-productionist STS analyses to include ideas on what constitutes ‘nature’ in the formulation of environmental problems, and how actors frame the relation between nature, technology, and society in environmental transformations.

Although the compatibilities between the envirotechnical approach and STS are already well delineated (Pritchard, 2011, 2013; Rowland & Passoth, 2015), there might still some limitations to be considered. The difficulty of a cross-disciplinary theorizing commonly stems from the respective ontological and epistemological bases of STS and environmental history. Simultaneously opening the black boxes of nature and technology may prove challenging, opening up the risk of turns to determinism or realism (Pritchard, 2011).

In the end, the envirotechnical approach is but one of many tools that STS analyses may use in order to make sense of alternative visions and their stabilizations within governance of sustainable environmental futures. Envirotechnical analysis can be further refined to offer an appropriate set of conceptual tools for STS research that has an interest in environmental transformations and alternatives to it. In times of accentuated expressions about global environmental change, bringing together perspectives from environmental history and STS may become increasingly important to understand the relationships between environmental knowledge, governance, and power within the enactment of environmentally transformative practices.
